# Acid-base disorders according to the Stewart approach in septic patients

**DOI:** 10.1186/cc13619

**Published:** 2014-03-17

**Authors:** J Szrama, P Smuszkiewicz, I Trojanowska

**Affiliations:** 1 University Hospital, Poznan, Poland

## Introduction

ICU patients often develop acid-base disorders. In clinical practice, there are several methods (for example, Henderson- Hasselbalch) used to interpret acid-base data, but most of them provide little information and fail to identify the cause of the problem. Stewart's approach which is based on physicochemical principles has growing popularity among clinicians in critical care. This method includes three independent variables that determine plasma pH (strong ion difference (SID), PCO_2_, and total weak acid concentration - mainly albumins and phosphate).

## Methods

The prospective analysis of arterial blood gases (ABG) was performed according to the Henderson-Hasselbalch approach and the Stewart method. The results were categorized into three groups according to the Henderson-Hasselbalch concept and the BE values: BE <-2, metabolic acidosis; BE between -2 and 2, normal values; BE >2, metabolic alkalosis. The aim of the study was to compare the efficacy of the traditional Henderson-Hasselbalch approach with acid-base disturbances with the Stewart concept in the population of critically ill septic patients.

## Results

The analysis included 990 arterial blood gases taken from 43 consecutive septic patients admitted to the ICU. One hundred and ninety-three ABG results met the criteria of metabolic acidosis, 473 were categorized into the metabolic alkalosis group and 324 results were within the range value of BE according to the Henderson-Hasselbalch concept. In the metabolic acidosis group (BE <-2), 34.7% of the results had elevated lactate concentration, 100% revealed hypoalbuminemia, 96.9% had Cl/Na ratio >0.75 revealing SID acidosis, while 42.5% met the criteria of SIG acidosis. In the normal range BE group, 21.3% revealed lactate concentration >2 mmol/l, 100% had hypoalbuminemia, 98.4% had Cl/Na ratio >0.75 revealing SID acidosis and hyperchloremia, while 14.5% showed SIG acidosis. The analysis of the aBg with BE >2 group showed that 18.4% had elevated lactate concentration, 99.1% revealed hypoalbuminemia, 88.8% had Cl/Na ratio >0.75 (SID acidosis) and 4.6% showed SIG acidosis.

## Conclusion

In critically ill patients with the BE values <-2, with BE in the range of normal value and BE >2 we observed complex acid-base disturbances with coexistence of hyperchloremic acidosis, hyperlactatemia, SIG acidosis and alkalosis caused by hypoalbuminemia. The Stewart approach is more effective in detecting acid-base disturbances and quantifying individual components of acid-base abnormalities and provides a detailed insight into their pathogenesis.

